# Phylogeny of *Saxifraga* section *Saxifraga* subsection *Arachnoideae* (Saxifragaceae) and the origin of low elevation shade‐dwelling species

**DOI:** 10.1002/ece3.9728

**Published:** 2023-01-09

**Authors:** Michael A. Gerschwitz‐Eidt, Markus S. Dillenberger, Joachim W. Kadereit

**Affiliations:** ^1^ Institut für Organismische und Molekulare Evolutionsbiologie, Johannes Gutenberg‐Universität Mainz Germany; ^2^ Institut für Biologie, AG Systematische Botanik und Pflanzengeographie, Freie Universität Berlin Berlin Germany; ^3^ Present address: Systematik, Biodiversität und Evolution der Pflanzen Ludwig‐Maximilians‐Universität München Munich Germany

**Keywords:** adaptive evolution, Alps, Hybseq, indicator values, quaternary, rear edge

## Abstract

*Saxifraga* section *Saxifraga* subsection *Arachnoideae* is a lineage of 12 species distributed mainly in the European Alps. It is unusual in terms of ecological diversification by containing both high elevation species from exposed alpine habitats and low elevation species from shady habitats such as overhanging rocks and cave entrances. Our aims are to explore which of these habitat types is ancestral, and to identify the possible drivers of this remarkable ecological diversification. Using a Hybseq DNA‐sequencing approach and a complete species sample we reconstructed and dated the phylogeny of subsection *Arachnoideae*. Using Landolt indicator values, this phylogenetic tree was used for the reconstruction of the evolution of temperature, light and soil pH requirements in this lineage. Diversification of subsection *Arachnoideae* started in the late Pliocene and continued through the Pleistocene. Both diversification among and within clades was largely allopatric, and species from shady habitats with low light requirements are distributed in well‐known refugia. We hypothesize that low light requirements evolved when species persisting in cold‐stage refugia were forced into marginal habitats by more competitive warm‐stage vegetation. While we do not claim that such competition resulted in speciation, it very likely resulted in adaptive evolution.

## Graphical abstract

*Saxifraga* sect. *Saxifraga* subsect. Arachnoideae is unusual in terms of ecological diversification by containing both high elevation species from exposed alpine habitats and low elevation species from shady habitats. Based on phylogenetic relationships, the geographical distribution of species in relation to refugial areas in the Alps and a reconstruction of ecological preferences we hypothesize that low light requirements evolved when species persisting in cold‐stage refugia were forced into marginal habitats by more competitive warm‐stage vegetation.

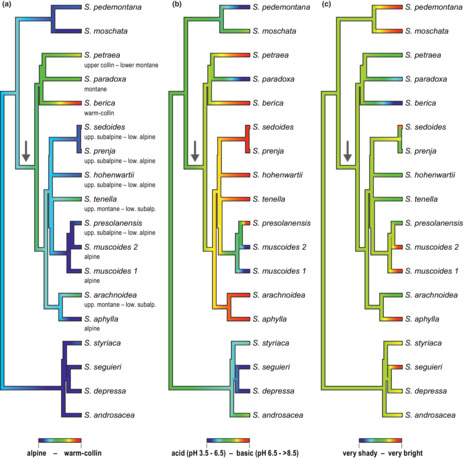

## 1 INTRODUCTION

The response of plants to the climatic oscillations of the Quaternary were extinction, migration or evolution (Bennett, 1997). Whereas extinction of species was rare but has been documented (Bennett, 1997), migration into and out of refugial areas has been amply documented based on fossil evidence, particularly pollen fossils (Bennett, 1997; Birks, 2019; Lang, 1994), phylogeography (Hewitt, 1996, 2000, 2004), the analysis of ancient DNA (Birks & Birks, 2016) and species distribution modeling (Svenning et al., 2011). Although it has been argued that periods of isolation required for speciation were never long enough in the Quaternary (Willis & Niklas, 2004), the climatic oscillations of the Quaternary have been shown to have resulted in evolution, i.e., genetic differentiation and speciation, often by changes in geographical distribution resulting in range fragmentation and divergence in geographical isolation, but also by hybrid speciation upon secondary contact (Kadereit & Abbott, 2022).

In Europe, the distribution of intraspecific genetic variation, and also of regional endemics, in combination with geological and palaeoenvironmental evidence, have been used to identify major refugial areas for, e.g., alpine species in and around the Alps (Schönswetter et al., 2005; Tribsch & Schönswetter, 2003), and for the Mediterranean area (Hewitt, 2011; Médail & Diadema, 2009; Nieto Feliner, 2011). Environmental conditions in areas which served as glacial refugia were not the same as in those areas in which species went extinct in glacial periods of the Quaternary (Bennett, 1997; Davis & Shaw, 2001; Hewitt, 1996, 2000, 2004). Thus, refugia clearly were not simply sanctuaries where species were preserved from extinction (Nieto Feliner, 2011), but adaptation to different conditions in these glacial refugia may have resulted in genetic divergence and eventually speciation (Davis & Shaw, 2001; De Lafontaine et al., 2018; Hewitt, 1996, 2000; Stewart et al., 2010; Stewart & Stringer, 2012). Moreover, abiotic and biotic conditions in areas serving as refugia clearly were subject to changes through Quaternary times. Considering alpine species, the glacial refugial areas in and around the European Alps identified by Schönswetter et al. (2005) were cold‐stage refugia (Birks & Willis, 2008). As shown by fossil evidence, many alpine species were much more widespread outside these refugia in glacial times (Birks & Willis, 2008; Tzedakis et al., 2013). In Quaternary interglacials and the Holocene, both changing climatic conditions and increasing competition resulted in the extant ranges of most alpine species, which can be considered interglacial (Bennett & Provan, 2008) or warm‐stage refugia (Bhagwat & Willis, 2008; Birks & Willis, 2008). However, populations of alpine species also persisted in mostly small areas within the former cold‐stage refugial area in habitats unsuitable for more competitive components of warm‐stage vegetation (Birks & Willis, 2008; Gentili et al., 2015; Pigott & Walters, 1954). Such persisting populations or species experienced dramatic changes in environmental conditions, from cold‐stage to warm stage‐conditions, through time, and these changes have driven evolutionary divergence in some cases (e.g., Scheepens et al., 2013).

**FIGURE 1:**
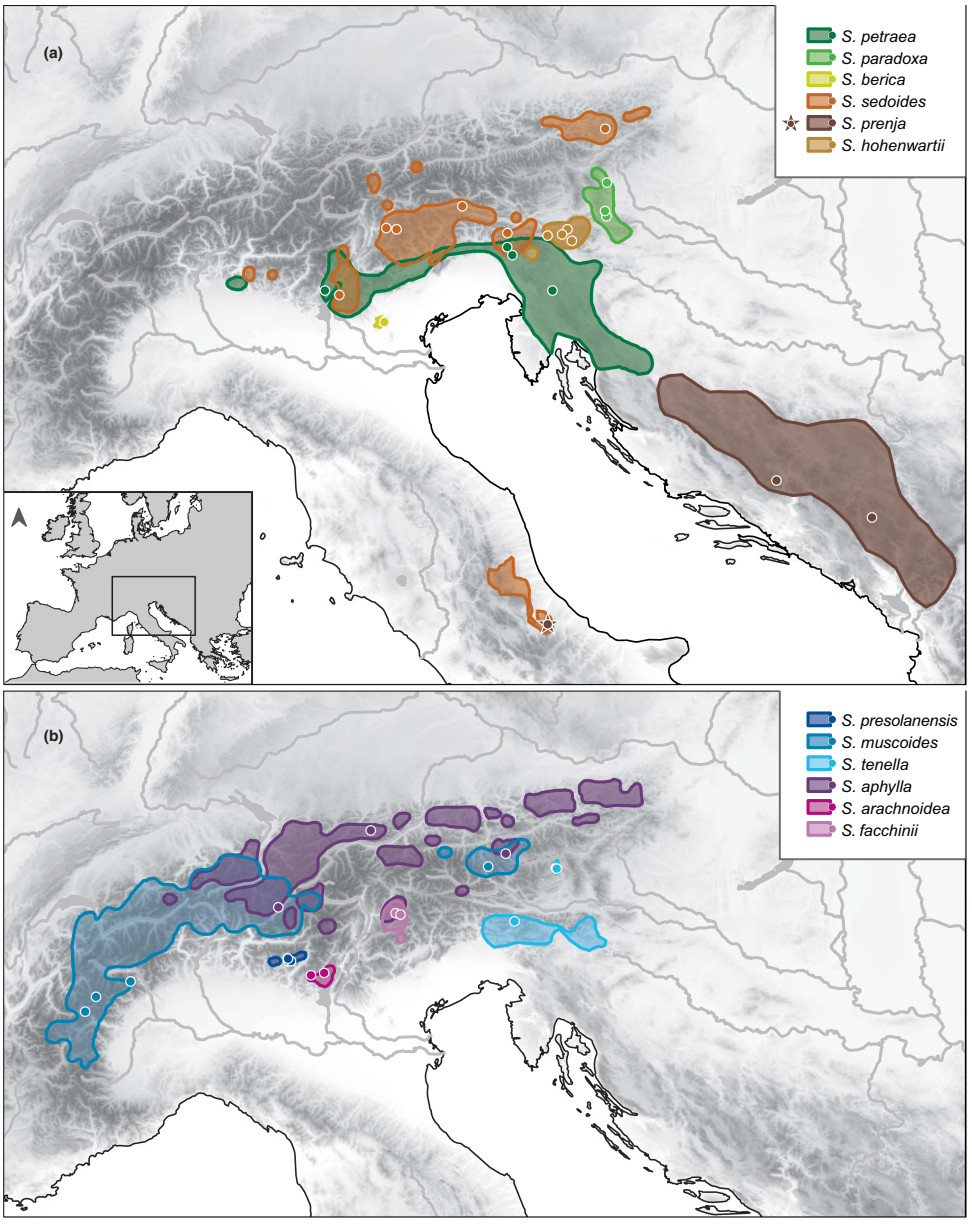
Distribution of the species of *Saxifraga* subsection *Arachnoideae* based on Kaplan (1995). The brown asterisk indicates a disjunct population of *S. prenja* in the Apennines as found by Gerschwitz‐Eidt and Kadereit (2020). For clarity, distribution ranges are shown in two maps (a, b). The locations of samples analyzed are indicated by dots.

*Saxifraga* L. section *Saxifraga* subsection *Arachnoideae* (Engl. & Irmsch.) Tkach, Röser & M.H.Hoffm. was first recognized by Tkach et al. (2015), and currently comprises 12 species (Ebersbach et al., 2017; Gerschwitz‐Eidt & Kadereit, 2020; Tkach et al., 2019). These are *S. aphylla* Sternb., *S. arachnoidea* Sternb., *S. berica* (Bég.) D.A.Webb, *S. facchinii* W.D.J.Koch, *S. hohenwartii* Vest ex Sternb., *S. muscoides* All., *S. paradoxa* Sternb., *S. petraea* L., *S. prenja* Beck, *S. presolanensis* Engl., *S. sedoides* L. and *S. tenella* Wulfen. Apart from *S. prenja* mainly from the Balkans, and a disjunct subrange of *S. sedoides* in the Apennines, subsection *Arachnoideae* occurs only in or near the Alps (Figure 1). The subsection is well known ecologically (Kaplan, 1995; Landolt et al., 2010; Webb & Gornall, 1989) and is most remarkable in terms of ecological diversification. While, e.g., the calcifuge *S. muscoides* grows largely above the tree‐line at elevations of up to 4200 m, the calcicole *S. berica* is limited to a small area in the Colli Berici near Vicenza (northern Italy) outside the Alps where it grows in shady hollows under overhanging rocks at elevations lower than 450 m. Other species of the subsection mostly growing in very shady and humid conditions under overhanging rocks, in recesses and hollows or at the entrance of caves, mostly at lower than alpine and frequently at collin or montane elevations, are *S. arachnoidea* and *S. paradoxa*, which have often been interpreted as Tertiary relics in the past (Gams, 1933; Meusel, 1943; Pitschmann & Reisigl, 1959; von Hayek, 1908).

Published phylogenetic analyses of subsection *Arachnoideae* either did not include all taxa and did not succeed in fully resolving phylogenetic relationships (Ebersbach et al., 2017; Tkach et al., 2015, 2019), or, when sampling all species (Gerschwitz‐Eidt & Kadereit, 2020), did not succeed in resolving phylogenetic relationships and identified supported conflict between nuclear and plastid phylogenetic trees. Against this background, we here aim at reconstructing and dating the phylogeny of subsection *Arachnoideae* with a Hybseq approach using a bait set of 329 protein‐coding nuclear loci designed for phylogenetic reconstructions in Saxifragales (Folk et al., 2019; Stubbs et al., 2018). This phylogenetic tree will be used to explore the ancestral ecology of the subsection. It seems possible that either species from shady conditions and mostly low elevations (*S. arachnoidea*, *S. berica*, *S. paradoxa*) or species from bright habitats at high elevations represent the ancestral habitat. We will also examine whether interspecific hybridization affected the evolution of ecological preferences in the group. Considering phylogenetic relationships, extant ecology and geographical distribution in relation to the location of known refugia we finally aim at identifying possible drivers of ecological diversification in the lineage.

## 2 MATERIALS AND METHODS

### 2.1 Sampling and DNA sequencing

Altogether 41 samples of all 12 species of *Saxifraga* section *Saxifraga* subsection *Arachnoideae*, one sample each of all five species of *Saxifraga* section *Saxifraga* subsection *Androsaceae* (Engl. & Irmsch.) Tkach, Röser & M.H.Hoffm. and one sample of *Saxifraga irrigua* M.Bieb., the last two very close relatives of subsection *Arachnoideae*, were collected from wild populations in the European Alps, the Apennines and the Dinaric Alps in 2016 or were obtained from herbarium material (Table 1). DNA was extracted from dried specimens using a Macherey‐Nagel NucleoSpin Plant II kit (Machery‐Nagel GmbH & Co. KG, Düren, Germany) and purified by ethanol precipitation following Sambrook and Russell (2001). For creating a next generation sequencing (NGS) DNA library, 400–2000 ng of DNA per sample were used. The libraries were pooled in groups of five to seven individuals and enriched using hybrid capture‐based target enrichment (Lemmon et al., 2012; Lemmon & Lemmon, 2013) for a panel of 301 loci (Folk et al., 2019) plus additional 28 loci from Stubbs et al. (2018). The enriched NGS DNA libraries were mixed with 10% of non‐enriched DNA library and sequenced on an Illumina HiSeq 2500 sequencer with a read length of 2 × 250 bp and a total number of 2 × 138 M reads. Library construction, target enrichment, and DNA sequencing were performed by Arbor Biosciences (Ann Arbor, Michigan, USA). All DNA sequences generated in this study were submitted to the NCBI short‐read archive (Table 1). Additionally, DNA sequences of 17 species of *Saxifraga* subsection *Saxifraga* (sensu Tkach et al., 2015) and of *Saxifraga hirsuta* L. of *Saxifraga* section *Gymnopera* D.Don were added from the NCBI BioProject PRJNA492276 (Folk et al., 2019).

**TABLE 1:** Sampling information

Classification (sensu Tkach et al., 2015)	Taxon	Sample	Accession type	Coll. Area; collector and year; coll. no.; herbarium no.	SRA accession no.
*Saxifraga* sect*. Gymnopera*	*S. hirsuta*	SAMN10066208	NCBI sra	n. a.; n. a.; n. a.; n. a.	SRR7901338
*S*. sect*. Saxifraga*	*S. irrigua*	GJO 0071452	Herbarium	Ukraine, Autonomous Republic of Crimea, Crimean mountains; Karl 2013; n. a.; GJO 0071452	*SRR13754157*
*S*. subsection *Androsaceae*	*S. androsacea*	84	Herbarium	Germany, Bavaria, Bavarian Alps; Dörr 1984; n. a.; M 0216596	*SRR13754166*
	*S. depressa*	209	Fresh material	Italy, Trentino‐South Tyrol, Dolomites; Gerschwitz‐Eidt 2016; MG16072012, MJG 022182	*SRR13754120*
	*S. italica*	203	Fresh material	Italy, Abruzzo, Gran Sasso d'Italia; Gerschwitz‐Eidt 2016; MG16073114; MJG 022204	*SRR13754156*
	*S. seguieri*	27	Herbarium	Italy, Trentino‐South Tyrol, Western Rhaetian Alps; Doppelbaur & Doppelbauer 1967; 17322; M 0216661	*SRR13754129*
	*S. styriaca*	205	Fresh material	Austria, Styria, Lower Tauern; Gerschwitz‐Eidt 2016; MG16053014; MJG 022249	*SRR13754128*
*S*. subsection *Arachnoideae*
	*S. aphylla*	MJG 022187	Fresh material	Switzerland, Grisons, Albula Alps; Gerschwitz‐Eidt 2016; MG16080223; MJG 022187	*SRR13754154*
	*S. aphylla*	LZ 163259	Herbarium	Germany, Bavaria, Bavarian Alps; Krusche 2000; 135/2000; LZ 163259	*SRR13754165*
	*S. aphylla*	WU 10832	Herbarium	Austria, Salzburg, Lower Tauern; Schönswetter & Tribsch 2005; 10832a; WU 18–38/4	*SRR13754132*
	*S. aphylla*	WU 18–38/4	Herbarium	Austria, Salzburg, Lower Tauern; Schönswetter & Tribsch 2005; 10832b; WU 18–38/4	*SRR13754143*
	*S. arachnoidea*	MJG 022191	Fresh material	Italy, Lombardy, Brescia and Garda Prealps; Gerschwitz‐Eidt 2016; MG16072311MG16072611, MJG 022191	*SRR13754124*
	*S. arachnoidea*	MJG 022199	Fresh material	Italy, Trentino‐South Tyrol, Brescia and Garda Prealps; Gerschwitz‐Eidt 2016; MG16072311, MJG 022199	*SRR13754123*
	*S. berica*	MJG 022246	Fresh material	Italy, Veneto, Colli Berici; Gerschwitz‐Eidt 2016; MG16060115; MJG 022246	*SRR13754121*
	*S. berica*	LZ 224384	Herbarium	Italy, Veneto, Colli Berici; Ebersbach 2017; Ebersbach 1458; LZ 224384	*SRR13754122*
	*S. facchinii*	MJG 022198	Fresh material	Italy, Trentino‐South Tyrol, Dolomites; Gerschwitz‐Eidt 2016; MG1672215; MJG 022198	*SRR13754164*
	*S. facchinii*	WU 18–38/8	Herbarium	Italy, Trentino‐South Tyrol, Dolomites; Hörandl 2006; Hörandl 9572; WU 18‐38/8	*SRR13754163*
	*S. hohenwartii*	MJG 025627	Fresh material	Austria, Carinthia, Carinthian–Slovenian Alps; Gerschwitz‐Eidt 2016; MG16071421; MJG 025627	*SRR13754161*
	*S. hohenwartii*	LZ 162908	Herbarium	Austria, Carinthia, Carinthian–Slovenian Alps; Gutte 2000; Gutte 252/2000; LZ 162908	*SRR13754162*
	*S. hohenwartii*	WU 18–38/12	Herbarium	Austria, Carinthia, Carinthian–Slovenian Alps; Hörandl & Hadaček 1991; 2521; WU 18–38/12	*SRR13754159*
	*S. hohenwartii*	WU 18–38/13	Herbarium	Austria, Carinthia, Carinthian–Slovenian Alps; Hörandl & Hadaček 1991; 2541; WU 18=38/13	*SRR13754158*
	*S. hohenwartii*	WU 18–38/9	Herbarium	Austria, Carinthia, Carinthian–Slovenian Alps; Hörandl & Hadaček 1992; Hörandl 4101; WU 18‐38/9	*SRR13754160*
	*S. muscoides*	GJO 0081449	Herbarium	Austria, Carinthia, Western Tauern Alps; Ocepek 2011; n. a.; GJO_0081449	*SRR13754155*
	*S. muscoides*	WU 029048	Herbarium	France, Auvergne–Rhône‐Alpes, Graian Alps; Schönswetter & Tribsch 2000; P‐13874‐Bio 4748; WU 029048	*SRR13754152*
	*S. muscoides*	WU 029049	Herbarium	Italy, Aosta valley, Graian Alps; Schönswetter & Tribsch 2000; P‐ 13874‐Bio 4806; WU 029049	*SRR13754153*
	*S. muscoides*	WU 18–38/16	Herbarium	France, Auvergne–Rhône‐Alpes, Graian Alps; Schneeweiß et al. 1998; Gutermann & al. 33417; WU 18–38/16	*SRR13754151*
	*S. paradoxa*	MJG 022203	Fresh material	Austria, Styria, Styrian Prealps; Gerschwitz‐Eidt 2016; MG16071611; MJG 022203	*SRR13754148*
	*S. paradoxa*	GJO 25642/11	Herbarium	Slovenia, Styrian Prealps; Melzer 1985; n. a.; GJO 25642/11	*SRR13754150*
	*S. paradoxa*	GJO 47231	Herbarium	Austria, Styria, Styrian Prealps; Bregant & Melzer 1981; n.a.; GJO 47231	*SRR13754149*
*S. petraea*	MJG 022190	Fresh material	Slovenia, Julian Alps and Prealps; Gerschwitz‐Eidt 2016; MG16071911; MJG 022190	*SRR13754144*

	*S. petraea*	MJG 022250	Fresh material	Slovenia, Julian Alps and Prealps; Gerschwitz‐Eidt 2016; MG16053112; MJG 022250	*SRR13754146*
	*S. petraea*	MJG 023532	Fresh material	Italy, Lombardy, Brescia and Garda Prealps; Gerschwitz‐Eidt 2016; MG16072717; MJG 023532	*SRR13754145*
	*S. petraea*	GJO 25642/104	Herbarium	Slovenia, Dinaric Alps; Melzer 1980 s; n. a.; GJO 25642/10	*SRR13754147*
	*S. prenja*	MJG 022200	Fresh material	Italy, Abruzzo, Maiella; Gerschwitz‐ Eidt 2016; MG16072937; MJG 022200	*SRR13754139*
	*S. prenja*	WU 18–38/25	Herbarium	Bosnia and Herzegovina, Federation of Bosnia and Herzegovina, Dinaric Alps; Schönswetter & Surina & al. 2007; W. Gutermann 38,835; WU 18–38/25	*SRR13754138*
	*S. prenja*	WU 18–38/26	Herbarium	Bosnia and Herzegovina, Republika Srpska, Dinaric Alps; Schönswetter & Surina & al. 2007; W. Gutermann 38,607; WU 18–38/26	*SRR13754137*
	*S. presolanensis*	MJG 022197	Fresh material	Italy, Lombardy, Brescia and Garda Prealps; Gerschwitz‐Eidt 2016; MG16072512; MJG 022197	*SRR13754141*
	*S. presolanensis*	GJO 25582/8	Herbarium	Italy, Lombardy, Bergamasque Alps and Prealps; Melzer 1984; n. a.; GJO 25582/8	*SRR13754142*
	*S. presolanensis*	WU 029320	Herbarium	Italy, Lombardy, Bergamasque Alps and Prealps; Schneeweiß & Schönswetter 2003; 8945; WU 0029320	*SRR13754140*
	*S. sedoides*	MJG 022196	Fresh material	Italy, Trentino‐South Tyrol, Dolomites; Gerschwitz‐Eidt 2016; MG16072111; MJG 022196	*SRR13754133*
	*S. sedoides*	MJG 025628	Fresh material	Italy, Friuli Venezia Giulia, Julian Alps and Prealps; Gerschwitz‐Edit 2016; MG16071723; MJG 025628	*SRR13754131*
	*S. sedoides*	GJO 0043514	Herbarium	Austria, Tyrol, Carnic and Gailtal Alps; Ster 2007; n. a.; GJO 0043514	*SRR13754136*
	*S. sedoides*	GJO 0088872	Herbarium	Austria, Styria, Northern Styrian Alps; Ocepek 2014; n. a.; GJO 0088872	*SRR13754135*
	*S. sedoides*	LZ 046270	Herbarium	Italy, Trentino‐South Tyrol, Dolomites; Gutte & Menhofer 1991; 5315; LZ 046270	*SRR13754134*
	*S. sedoides*	WU 18–38/32	Herbarium	Italy, Trentino‐South Tyrol, Brescia and Garda Prealps; Hörandl & Hadaček 1992; Hörandl 4591; WU 18–38/32	*SRR13754130*
	*S. tenella*	GJO 0081439	Herbarium	Austria, Carinthia, Carinthian‐Styrian Alps; Ocepek 2011; n. a.; GJO 0081439	*SRR13754126*
	*S. tenella*	GJO 26592/12	Herbarium	Austria, Carinthia, Carinthian‐Styrian Alps; Karl 1997; n. a.; GJO 26592/12	*SRR13754127*
*S. tenella*	MA 777464	Herbarium	Slovenia, Julian Alps and Prealps; Hörandl et al. 1993; Hörandl 5339; MA 777464	*SRR13754125*
*S*. subsection *Saxifraga*	*S. adscendens*	SAMN10066150	NCBI sra	n. a.; n. a.; n. a.; n. a.	SRR7901359
	*S. canaliculata*	SAMN10066165	NCBI sra	n. a.; n. a.; n. a.; n. a.	SRR7901413
	*S. cebennensis*	SAMN10066169	NCBI sra	n. a.; n. a.; n. a.; n. a.	SRR7901761
	*S. conifera*	SAMN10066177	NCBI sra	n. a.; n. a.; n. a.; n. a.	SRR7901613
	*S. continentalis*	SAMN10066179	NCBI sra	n. a.; n. a.; n. a.; n. a.	SRR7901611
	*S. corbariensis*	SAMN10066175	NCBI sra	n. a.; n. a.; n. a.; n. a.	SRR7901525
	*S. exarata*	SAMN10066190	NCBI sra	n. a.; n. a.; n. a.; n. a.	SRR7901478
	*S. fragilis*	SAMN10066199	NCBI sra	n. a.; n. a.; n. a.; n. a.	SRR7901318
	*S. globulifera*	SAMN10066204	NCBI sra	n. a.; n. a.; n. a.; n. a.	SRR7901350
	*S. humilis*	SAMN10066212	NCBI sra	n. a.; n. a.; n. a.; n. a.	SRR7901466
	*S. latepetiolata*	SAMN10066223	NCBI sra	n. a.; n. a.; n. a.; n. a.	SRR7901697

	*S. magellanica*	SAMN10066230	NCBI sra	n. a.; n. a.; n. a.; n. a.	SRR7901512
	*S. moschata*	SAMN10066239	NCBI sra	n. a.; n. a.; n. a.; n. a.	SRR7901734
	*S. pedemontana*	SAMN10066248	NCBI sra	n. a.; n. a.; n. a.; n. a.	SRR7901366
	*S. portosanctana*	SAMN10066254	NCBI sra	n. a.; n. a.; n. a.; n. a.	SRR7901409
	*S. pubescens*	SAMN10066256	NCBI sra	n. a.; n. a.; n. a.; n. a.	SRR7901600
*S. rosacea*	SAMN10066259	NCBI sra	n. a.; n. a.; n. a.; n. a.	SRR7901234

*Note*: SRA accession numbers in italics were newly generated in this study.

### 2.2 Sequence assembly and alignment

We merged forward and reverse reads for each sample and removed PCR duplicates using ParDRe v2.2.5 (González‐Domínguez & Schmidt, 2016). Adapter sequences as well as read segments of poor quality were removed with Trimmomatic v0.36 (Bolger et al., 2014) using a sliding window of 4 bp with a sliding window minimum phred quality score of 20. DNA sequence assembly was performed using the BWA version of HybPiper v1.3.1 (Johnson et al., 2016) with default settings. Exons were filtered for paralogs (Method 1 in Appendix A) and aligned across all samples locus‐wise using MAFFT v7.305 (Katoh & Standley, 2013). Seven species of *Saxifraga* subsection *Saxifraga* aligned poorly in most loci with high numbers of base mismatches and indels. We removed these samples from the data set and repeated the alignment of the exons with the 58 remaining samples. Subsequently, the non‐exonic sequences were added to the exonic alignments using the ‘‐addlong’ option in MAFFT. The resulting alignments frequently contained indel‐rich stretches of poorly aligned non‐exonic sequences which were several thousand base pairs long. We used BuddySuite v1.3.0 (Bond et al., 2017) to reduce the number of these regions by trimming alignment positions that consisted of more than 50% gaps. Any alignments that were missing more than 20% of the samples or the *S. hirsuta* sample were also excluded from further analysis. We removed residual paralogs with TreeShrink v1.3.3 (Mai & Mirarab, 2018; Method 2 in Appendix A). The resulting data set consisted of 405 loci in 58 samples.

### 2.3 Phylogenetic analysis

We calculated bootstrapped maximum likelihood (ML) gene trees for all 405 loci of the full data set in RAxML v8.2.12 (Stamatakis, 2014) and used ASTRAL v5.7.3 (Zhang et al., 2018) to infer two bootstrapped summary coalescent (SC) species trees from the 405 ML gene trees, using a taxon map for the second run (Rabiee et al., 2019). Taking into account the results of the first ASTRAL run, the *S. muscoides* samples were coded into two groups (*S. muscoides* 1 and 2) for the taxon map of the second ASTRAL run.

Additionally, we inferred phylogenetic networks to include both incomplete lineage sorting (ILS) and reticulation in the modeling process under the multispecies network coalescent (Wen et al., 2016). We calculated SC species networks from the 405 gene trees under the maximum pseudo‐likelihood (MPL) method “InferNetwork_MPL” (Yu & Nakhleh, 2015) in PhyloNet v3.8.0 (Than et al., 2008; Wen et al., 2018), using a taxon map and a gene tree bootstrap threshold of 70. We computed 11 MPL networks with a number of hybrid nodes (K) ranging from zero to ten. Each network was inferred with 10 independent runs to optimize the pseudo‐likelihood. For each K the best network weighted by pseudo‐likelihood was identified. We inferred the major trees, i.e., the species trees underlying the networks, from the best networks for K = 1–10 to explore the commonalities of the network topologies. For this purpose, all minor edges were removed from the best networks using the “majorTree” command in PhyloNetworks v.0.11.0 (Solís‐Lemus et al., 2017) as implemented in Julia‐REPL v.1.2.0 (Bezanson et al., 2017). The major trees were visually compared to draw conclusions about the monophyly of *Saxifraga* subsection *Arachnoideae* and to identify its most likely sister clade. To assess the relative model fit, we plotted the log‐likelihoods of the best networks against their respective K.

To assess uncertainty in network inference for interspecific relationships in *Saxifraga* subsection *Arachnoideae*, we pruned the data set to include the 41 samples of subsection *Arachnoideae* and one sample of *S. pedemontana* All. as new outgroup (see results) and removed any alignments that were missing more than 20% of the samples or the outgroup sample, resulting in a total of 388 loci. We here used *S. pedemontana* as outgroup because this species, which is part of section *Saxifraga* subsection *Saxifraga*, is more closely related to subsection *Arachnoideae* than *S. hirsuta* which was used as outgroup in those analyses which sampled across section *Saxifraga*. We calculated bootstrapped ML gene trees in RAxML v8.2.12 and used ASTRAL v5.7.3 to infer an SC species tree with 100 bootstrap replicates from the 405 ML gene trees, using a taxon map. We used SNaQ (Solís‐Lemus & Ané, 2016) as implemented in PhyloNetworks v0.11.0 to calculate six SC species networks for zero to five hybrid nodes (H), assessed the relative model fit by the Log pseudo‐likelihood profile and performed 100 bootstrap replicates each for the best networks with H = 1 and H = 2 (Method 3 in Appendix A).

### 2.4 Divergence times estimation

Divergence times for *Saxifraga* subsection *Arachnoideae* were modeled on a multi‐labeled phylogenetic tree (Blanco‐Pastor et al., 2012; Huber et al., 2006; Pirie et al., 2009). The multi‐labeled species tree used was based on the ASTRAL species tree as calculated from the full data set. Based on the network reconstructions, a second parent lineage of the hybrid species *S. facchinii* was added to the ASTRAL species tree and the two lineages of *S. facchinii* were labeled ‘*S. facchinii 1*’ and ‘*S. facchinii 2*’. Of the full data set of 58 samples, we selected 10 loci each for which the ML gene trees could be unambiguously assigned to either of the two topologies contained in the multi‐labeled tree (Method 4 in Appendix A). The 20 associated DNA alignments were visually inspected in MEGA X v10.0.5 (Kumar et al., 2018) and manually re‐aligned to ensure overall good alignment quality. In the ten alignments used to reconstruct the lineage ‘*S. facchinii 1’*, sequences of the two samples of *S. facchinii* were labeled with the suffix ‘1’. Correspondingly, in the ten alignments used to reconstruct the lineage ‘*S. facchinii 2’*, *S. facchinii* sequences were labeled with the suffix ‘2’. Time‐calibrated phylogenetic trees were calculated with STARBEAST2 v0.15.5 (Ogilvie et al., 2017) as implemented in BEAST v2.6.3 (Bouckaert et al., 2019), using an uncorrelated lognormal relaxed molecular clock (Drummond et al., 2006) with a birth‐death speciation process (Gernhard, 2008; Stadler, 2009) and the nucleotide substitution model GTR + Γ. Based on the results of a molecular dating analysis of Saxifragales (Folk et al., 2019), a uniformly distributed root prior of 9.12–24.4 Ma was used for a secondary calibration of the *Saxifraga* section *Saxifraga* stem. Tree topology was restricted to the multiple‐labeled species tree both to prevent a bias in divergence time estimation due to convergence on alternative tree topologies and to reduce calculation time. All other parameters of the model were optimized in STARBEAST2. We set up four independent runs, each with 62 parallel adaptive Metropolis‐coupled Markov chains (Altekar et al., 2004; Müller & Bouckaert, 2020) and run them for a total of 450 million generations. Every 5000th generation was sampled. We used Tracer v.1.7.1 (Rambaut et al., 2018) to assess convergence and an effective sample size of at least 200 to identify an appropriate burn‐in for each run. The four runs were combined using LogCombiner v.2.6.3 (Drummond & Rambaut, 2007). We used TreeAnnotator v.2.6.3 (Drummond & Rambaut, 2007) to calculate a maximum clade credibility tree from the species tree distribution. Node heights of the maximum clade credibility tree were set to the medians of the respective node height distributions. The chronogram was plotted using the R package phyloch (Heibl, 2013) in R v.4.1.2 (R Core Team, 2021).

### 2.5 Ecological trait reconstruction

We reconstructed the evolution of three ecological traits of *Saxifraga* subsection *Arachnoideae* using the ecological indicator values (EIV) of Landolt et al. (2010). According to Silvertown et al. (2006), EIVs can be considered as numerical representations of ecological niche traits and therefore can be used to reconstruct ecological niche evolution in a phylogenetic framework (Prinzing et al., 2001; Silvertown et al., 2006). Because EIVs represent quasi‐cardinal numbers (Ellenberg, 1992), they can be directly translated into continuous trait states as long as the EIVs under consideration show little intraspecific variation. Values for the niche trait indicators temperature (T), light (L) and soil pH (R) were taken from Landolt et al. (2010). Since Landolt EIVs were not available for *S. prenja*, we collected and comparatively reviewed available information on the ecology of *S. prenja* from various sources (Hörandl, 1993; Kaplan, 1995; Webb & Gornall, 1989). We concluded that the ecological traits of *S. prenja* are comparable to those of *S. hohenwartii* and used the EIVs of the latter for the former.

All taxa for which no Landolt EIVs exist were pruned from the time‐calibrated multi‐labeled species in the R package ape v5.0 (Paradis & Schliep, 2019). The hybrid species *S. facchinii* was removed because its inclusion violates the model's essential assumption of a bifurcating tree. As two independent measures of the strength of phylogenetic signal, we calculated Blomberg's K (Blomberg et al., 2003) and Pagel's *λ* (Pagel, 1999) using the R package phytools v.0.7–90 (Revell, 2012). We used the R package geiger v2.0.7 (Pennell et al., 2014) to fit four models of ecological trait evolution to all three EIVs, i.e. (1) a simple Brownian motion model (BM; Felsenstein, 1973), (2) an Ornstein‐Uhlenbeck model (OU; Butler & King, 2004), (3) an early burst model (EB; Harmon et al., 2010), and (4) a white‐noise model. We calculated the corrected Akaike information criteria (AICc) weights (wi) from the AICc of the four models as a measure of the model's predictive power. Reconstruction of ancestral traits was performed for each of the three EIVs under the respective model with the largest AICc weight using the phytools ‘anc.ML’ function with 1 million iterations each. Finally, we projected the inferred traits onto the pruned species tree using the ‘contMap’ function of phytools.

We also used the Maximum Parsimony approach for the reconstruction of continuous characters implemented in Mesquite v3.70 (Maddison & Maddison, 2021). Landolt EIVs were reconstructed as continuous characters with the squared change assumption. For the analysis we used the same phylogenetic tree as above.

## 3 RESULTS

### 3.1 DNA assembly and alignment statistics

In the initial assembly, reads mapped to 326 of the original 329 reference loci. After three iterations of assembly, visual paralog assessment and reference file editing, reads were mapped successfully to 567 loci in the final assembly, of which 463 were retained for further analysis. After filtering for a minimum sample coverage of 80% and for loci that included a sequence of the outgroup sample *S. hirsuta*, the full data set contained the filtered alignments of 405 loci with a total length of approximately 1.33 Mbp, an average taxon coverage per locus of 53.5 of 58 samples, a mean alignment length of 3293 bp, a mean proportion of missing data of 18%, a mean proportion of variable alignment positions of 60%, and a mean proportion of parsimony informative alignment positions of 40%. The pruned data set (samples of subsection *Arachnoideae* and *S. pedemontana* as outgroup) contained 388 loci with a total length of approximately 1.28 Mbp, an average taxon coverage of 39.3 of 42 samples, an average alignment length of 3294 bp, an average proportion of missing data of 10%, an average proportion of variable alignment positions of 40%, and an average proportion of parsimony informative alignment positions of 20%.

### 3.2 Phylogenetic relationships and hybridization

**FIGURE 2:**
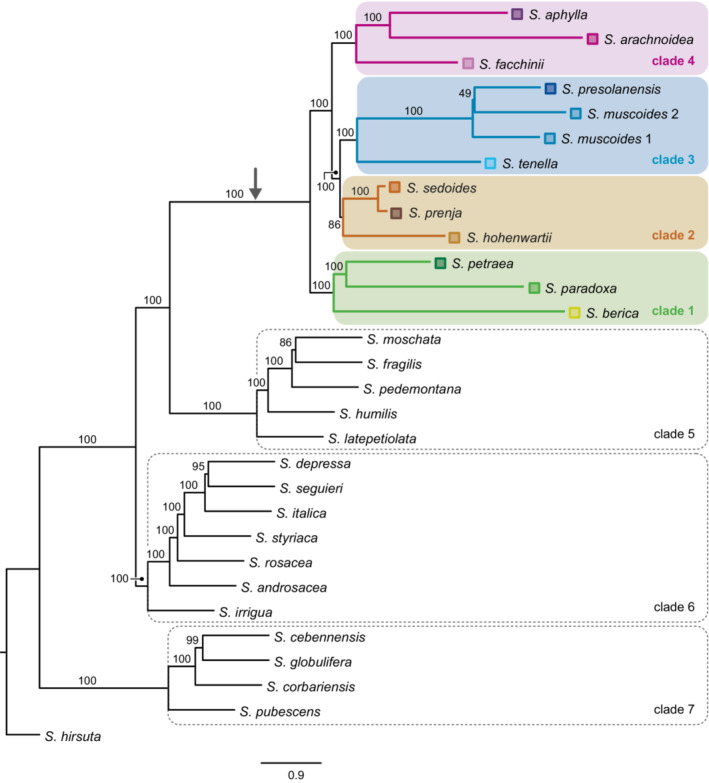
Summary coalescent species tree of *Saxifraga* subsection *Arachnoideae*. Branch lengths are shown in coalescent units. Numbers above branches are bootstrap support values. The stem branch of subsection *Arachnoideae* is marked with an arrow.

**FIGURE A1:**
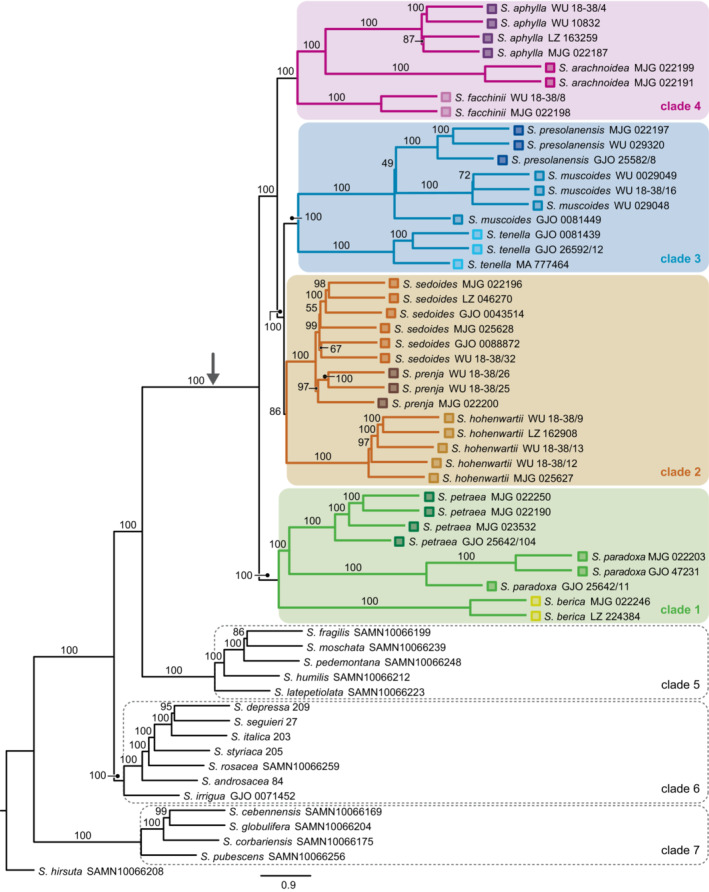
Cladograms of the major trees of 11 maximum pseudo‐likelihood phylogenetic networks calculated in PhyloNet. The stem branch of subsection *Arachnoideae* is marked with an arrow.

**FIGURE A2:**
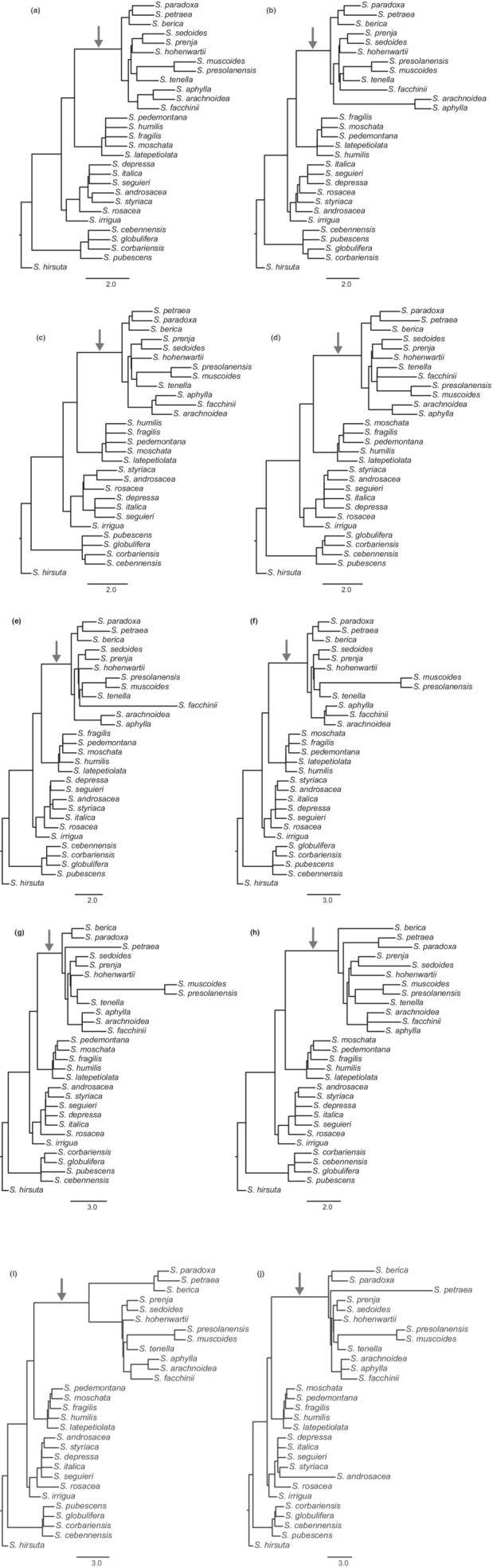
Log pseudo‐likelihood profiles for phylogenetic networks with (a) 58 and (b) 42 samples for different numbers of reticulate nodes.

For the full data set with 58 samples, the two ASTRAL species trees (Figure 2, Figure A1) and the major trees of the 10 best PhyloNet networks for K = 1 to K = 10 (Figure A2a–j) were mostly congruent. In all phylogenetic analyses, *Saxifraga* subsection *Arachnoideae* (sensu Gerschwitz‐Eidt & Kadereit, 2020) was reconstructed as monophyletic. In the ASTRAL trees, a clade of *S. berica*, *S. paradoxa* and *S. petraea* (clade 1) was sister to the rest of the group. In this, a clade of *S. hohenwartii*, *S. prenja* and *S. sedoides* (clade 2) was sister to a clade of *S. muscoides*, *S. presolanensis* and *S. tenella* (clade 3). A clade of *S. facchinii*, *S. aphylla* and *S. arachnoidea* (clade 4) was sister to clades 2 and 3.

**FIGURE A3:**
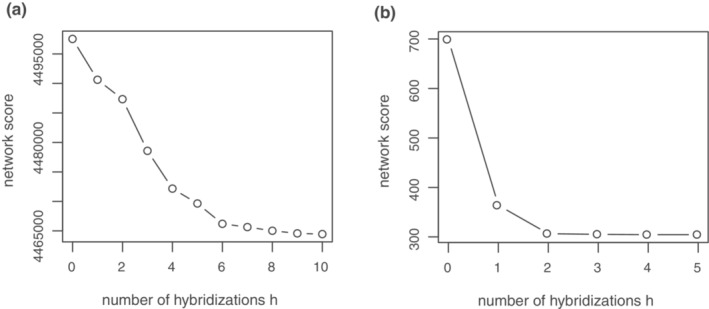
Summary coalescence species tree calculated in ASTRAL. The stem branch of subsection *Arachnoideae* is marked with an arrow.

Clades 1–4 were found in identical form only in the PhyloNet network for K = 1 (Figure A2a). In the PhyloNet networks for K = 2 to K = 10, reconstruction of clades 1–4 differed in the placement of only one or two taxa (Figure A2b–j). Three additional clades were always reconstructed: First, a clade consisting of *S. moschata* Wulfen, *S. fragilis* Schrank, *S. pedemontana*, *S. humilis* Engl. & Irmsch. and *S. latepetiolata* Willk. (clade 5) as sister to subsection *Arachnoideae* (clades 1–4). A clade of *Saxifraga* subsection *Androsaceae*, *S. irrigua* and *S. rosacea* Moench (clade 6) was identified as sister to clades 1–5. A clade consisting of *S. cebennensis* Rouy & E.G. Camus, *S. globulifera* Desf., *S. corbariensis* Timb.‐Lagr., and *S. pubescens* Pourr. (clade 7) was reconstructed as sister to clades 1–6. No optimal number of reticulations could be inferred unequivocally from the shape of the saturation curve of the log likelihoods of the phylogenetic networks for K = 0 to K = 10 (Figure A3a).

**FIGURE 3:**
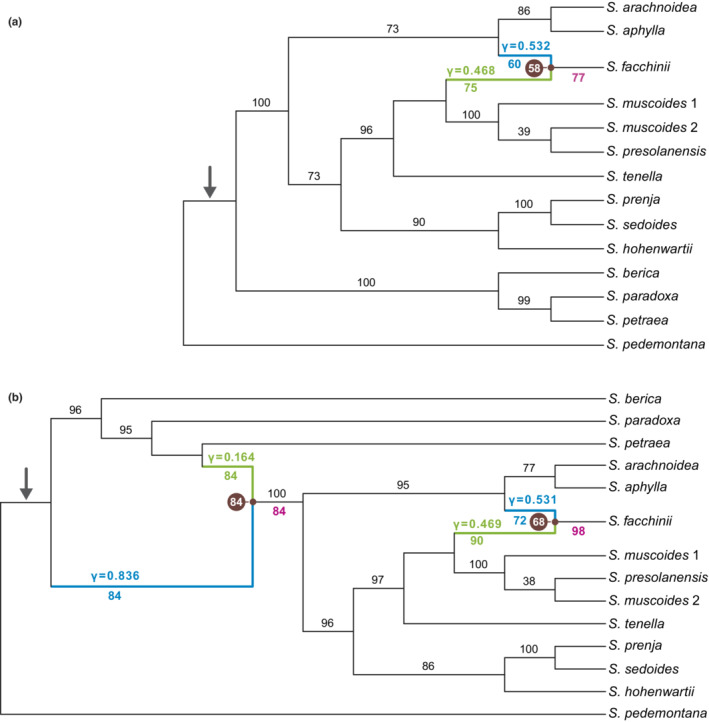
Summary coalescent species networks of *Saxifraga* subsection *Arachnoideae* modeled with (a) one and (b) two reticulation events. Branch lengths are non‐informative. Reticulation edges are indicated by blue lines for the major parent and green lines for the minor parent of a reticulation event. Numbers in blue or green below reticulation edges are inheritance probabilities. Numbers in black above branches are branch support values of regular edges. Numbers below a branch in magenta indicate a hybrid branch and are hybrid edge support values. Numbers in brown circles next to a network node are support values for that specific combination of two parental reticulation edges and a progeny hybrid edge. The stem branch of subsection *Arachnoideae* is marked with an arrow.

For the data set with 42 samples, the saturation curve of the pseudo‐likelihoods of the networks plotted against H showed a strong decrease from H = 0 to H = 1 and a moderate decrease from H = 1 to H = 2 (Figure A3b). A further increase of H resulted in only minor improvement of the pseudo‐likelihood, indicating that one or two hybrid nodes are the best hypotheses for the true species network topology. The ASTRAL species tree and the major trees of the species networks were congruent with each other. Consistent with the 58 samples data set ASTRAL species trees and the major trees of the phylonet species networks, clades 1–4 as described above were also found in the 42 samples species networks of subsection *Arachnoideae* (Figure 3a,b).

In the best networks for H = 1 (Figure 3a) and H = 2 (Figure 3b), *S. facchinii* was reconstructed as a hybrid species (BS = 77 and BS = 98, respectively). In 58 (H = 1) and 68 (H = 2) of 100 BS replicates, the hybrid parents were a lineage in clade 3 (BS = 75 and BS = 90, respectively) with inheritance values of γ = 0.468 and γ = 0.469, respectively, and a lineage in clade 4 (BS = 60 and BS = 72, respectively) with γ = 0.532 and γ = 0.531, respectively. A second hybrid node (BS = 84) was reconstructed in the network for H = 2, with one of the two hybrid edges connecting the stem of *S. petraea* (BS = 84, γ = 0.164) to the stem lineage of clades 2–4 (BS = 84, γ = 0.836).

### 3.3 Divergence times

**FIGURE 4:**
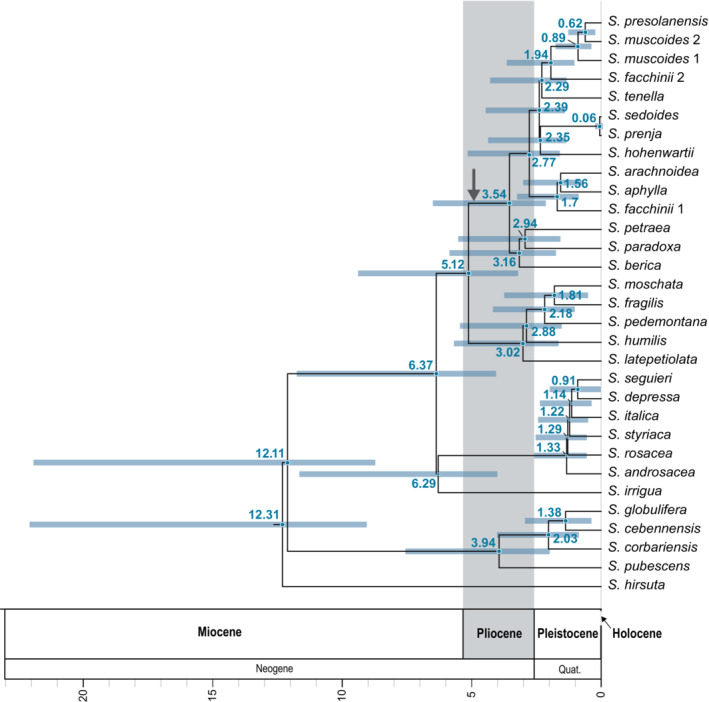
Chronogram of *Saxifraga* subsection *Arachnoideae*. Horizontal blue bars indicate 95% confidence intervals. Node numbers are median node ages in million years. The stem branch of subsection *Arachnoideae* is marked with an arrow.

For the first topology in the multi‐labeled phylogenetic species tree, with the parent lineage of *S. fachinii* in clade 4, we selected 10 alignments with a length of 40,970 bp. For the second topology, with the parent lineage of *S. fachinii* in clade 3, we selected 10 alignments with a length of 33,130 bp. The maximum clade credibility tree was constructed from a total of 133,204 dated species trees (Figure 4). The stem age of subsection *Arachnoideae* was dated to 5.12 (95% confidence interval 3.28–9.32) million years ago (myr). Their crown age was estimated at 3.54 (2.21–6.43) myr. For clade 1, stem and crown ages of 3.54 (2.21–6.43) myr and 3.16 (1.81–5.79) myr were estimated. Stem and crown ages of clade 4 were estimated at 2.77 (1.66–5.08) myr and 1.7 (0.94–3.18) myr, respectively. The stem age of the *S. facchinii* lineage in this clade corresponds to the clade crown age. For clade 2, stem and crown ages of 2.39 (1.47–4.39) myr and 2.35 (1.42–4.3) myr were estimated. In clade 2, the overall youngest node was found for the last common ancestor (MRCA) of *S. sedoides* and *S. prenja* with an age of 0.06 (0–0.16) myr. Stem and crown ages of 2.39 (1.47–4.39) myr and 2.29 (1.41–4.22) myr were calculated for clade 3. The stem age of the *S. facchinii* lineage in this clade was dated to 1.94 (1.1–3.57) myr.

### 3.4 Ancestral ecology

**FIGURE 5:**
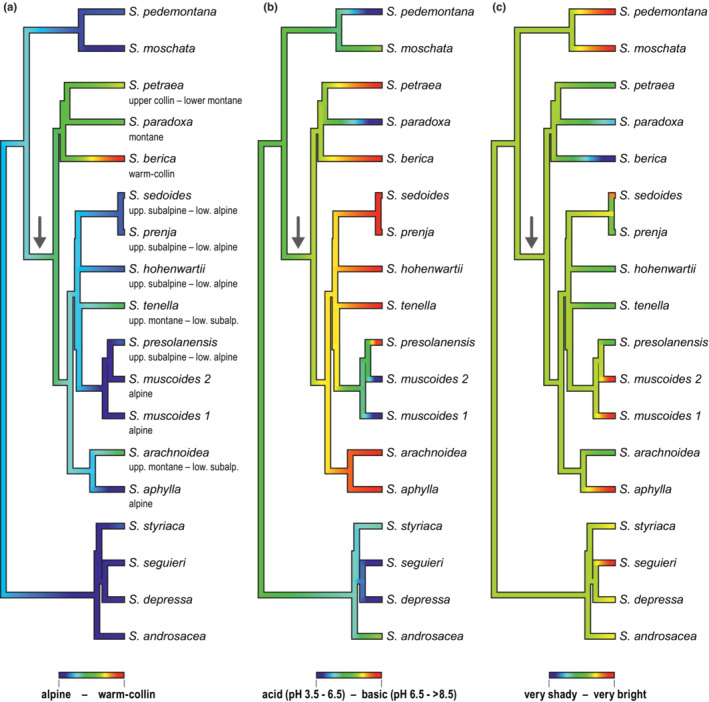
Ancestral state reconstructions in *Saxifraga* subsection *Arachnoideae* for (a) temperature, (b) soil pH and (c) light preferences. Indicator values for the stem group node of subsection *Arachnoideae* were reconstructed as 1.96/1.96 (Geiger/Mesquite; subalpine), 3.66/3.66 (neutral to slightly alkaline) and 3.77/3.76 (just below bright). Bars below phylogenetic trees explain the color scheme used. The stem branch of subsection *Arachnoideae* is marked with an arrow.

Based on Blomberg's K and Pagel's *λ*, a strong phylogenetic signal was detected in the temperature indicator (K = 0.947, *p* = .004; *λ* = 1.009, *p* = 8.01 × 10^−5^). Consistent with this finding, a simple Brownian motion model was fitted as the best model (wi = 0.642; Table 2). The stem group node of subsection *Arachnoideae* was reconstructed as 1.96, corresponding to subalpine elevations, and its descendants shifted into lower (clade 1) or higher elevations (clades 2–4, Figure 5a). The same result (1.96) was obtained in our Mesquite reconstruction. No phylogenetic signal was indicated for the light indicator (K = 0.120, *p* = .627; *λ* = 4.83 × 10^−5^, *p* = 1.0) and for the soil pH indicator (K = 0.448, *p* = .123; *λ* = 0.234, *p* = .494). Accordingly, white noise models were fitted as the best models for soil pH and light niche evolution (Table 2). The second best model for soil pH evolution was an OU model (α = 0.692, wi = 0.390). According to the model, the stem group node of subsection *Arachnoidea*e was reconstructed as 3.66, corresponding to neutral to slightly alkaline soils, and adaptation to base‐poor soils took place independently in *S. paradoxa* and the MRCA of *S. muscoides and S. presolanensis* (Figure 5b). The same result (3.66) was obtained in our Mesquite reconstruction. The second best model for light niche was also an OU model, however, with a large rubber band parameter α and a low Akaike weight (α = 321.974, wi = 0.189) indicating poor model fit. According to the model, the stem group node of subsection *Arachnoideae* was reconstructed as 3.77, corresponding to just below bright. Adaptation to half‐shady, shady or very shady habitats took place independently in *S. arachnoidea*, *S. paradoxa* and *S. berica*. The Mesquite reconstruction resulted in a very similar value (3.76).

**TABLE 2:** Statistics for tests of phylogenetic signal and model fit for Landolt ecological indicator values (EIV)

EIV	Phylogenetic signal						Model fit					
	Blomberg's K		Pagel's *λ*									
	K	*p*	*λ*	logL(*λ*)	LR (*λ* = 0)	*p*	Variable	BM	OU	EB	WN	Best model
Temperature (T)	0.947	.004	1.009	−17.567	15.556	8.01 × 10^−5^	a	–	–	−0.285	–	BM
							Alpha	–	0	–	–	
							sigsq	0.214	0.214	0.813	0.978	
							z0	1.807	1.806	1.740	1.778	
							Log‐likelihood	−20.073	−20.073	−19.754	−25.344	
							AIC	44.146	46.146	45.508	54.689	
							AICc	44.946	47.860	47.222	55.489	
							fDOF	2	3	3	2	
							AIC‐weight	0.642	0.149	0.206	0.003	
Light (L)	0.120	.627	4.83 × 10^−5^	−28.537	−1.04 × 10^−4^	1	a	–	–	−1 × 10^−6^	–	WN
							Alpha	–	321.974	–	–	
							sigsq	2.415	898.348	2.415	1.395	
							z0	3.844	3.778	3.844	3.778	
							Log‐likelihood	−41.875	−28.537	−41.875	−28.537	
							AIC	87.751	63.075	89.751	61.075	
							AICc	88.551	64.789	91.465	61.875	
							fDOF	2	3	3	2	
							AIC‐weight	1.31 × 10^−6^	0.189	3 × 10^−7^	0.811	
Reaction (R)	0.448	.123	0.234	−30.792	0.468	.494	a	–	–	−1 × 10^−6^	–	WN
							Alpha	–	0.692	–	–	
							sigsq	0.884	2.556	0.884	1.840	
							z0	3.514	3.809	3.514	3.778	
							Log‐likelihood	−32.829	−29.830	−32.829	−31.026	
							AIC	69.657	65.659	71.657	66.053	
							AICc	70.457	67.374	73.372	66.853	
							fDOF	2	3	3	2	
							AIC‐weight	0.084	0.390	0.019	0.507	

*Note*: Numbers have been rounded to the third decimal place.

Apart from our reconstruction of ecological traits, *S. arachnoidea* as a species from upper montane to lower subalpine elevations and shady to half‐shady habitats clearly differs strongly from its closest relative, *S. aphylla*, a species from alpine elevations and very bright habitats (Landolt et al., 2010). In case of *S. berica* and *S. paradoxa*, *S. paradoxa* grows on acid substrates in contrast to *S. berica* (basic) and *S. petraea* (basic). Differentiation in light requirements are less pronounced than in *S. arachnoidea*/*S. aphylla*, with *S. berica* growing in very shady, *S. paradoxa* in shady and *S. petraea* in half‐shady habitats (Landolt et al., 2010).

## 4 DISCUSSION

### 4.1 Phylogeny of subsection *Arachnoideae* and the origin of ecologically divergent species

The three species of clade 1, *S. petraea*, *S. paradoxa* and *S. berica* all grow in shady and humid conditions under overhanging rocks, in recesses and hollows or at the entrance of caves. Whereas *S. berica*, only known from the Colli Berici near Vicenza (northern Italy), grows at elevations lower than 450 m, both *S. petraea* and *S. paradoxa* mostly grow at elevations lower than 500 m but occasionally can be found at higher elevations (Kaplan, 1995; Webb & Gornall, 1989). Considering the extant distribution of the three species (Figure 1) and their relationships to each other (Figure 2), speciation likely was allopatric and the narrowly distributed *S. berica* split from the more widely distributed *S. petraea + S. paradoxa* before these two latter species separated. Following Landolt et al. (2010), ecological divergence in terms of light requirements is very limited in this clade (*S. berica*: very shady; *S. paradoxa*: shady; *S. petraea*: half‐shady). Interestingly, *S. paradoxa*, by growing on gneiss or mica‐schists, is one of only two species of the subsection which is not calcicole. The extant range of *S. paradoxa* lies in the ‘easternmost Central Alps’ refugium for calcifuge taxa (Schönswetter et al., 2005; Tribsch & Schönswetter, 2003), and that of *S. berica* in the ‘Monte Baldo, Monti Lessini, and Prealpi Bellunesi’ refugium for calcicole taxa (Schönswetter et al., 2005; Tribsch & Schönswetter, 2003). Of all clades of subsection *Arachnoideae* (except part of *S. sedoides* and *S. prenja* of clade 2, see below), clade 1 has the southernmost distribution range by occurring along the southern and eastern periphery of the Alps. As clade 1 is sister to the remainder of the subsection, it is likely that clade 1 as a whole separated parapatrically from the remainder of the subsection in a cold phase of the late Pliocene and acquired its extant ecology in its further evolution (see below). The hypothesis of an essentially parapatric divergence of clade 1 from the remainder of the subsection finds support in the detection of hybridization between *S. petraea* and the last common ancestor of clades 2, 3 and 4 (Figure 3b).

As in clade 1, the three species of clade 2, *S. sedoides*, *S. hohenwartii* und *S. prenja*, are largely allopatric in distribution. The only exception from this is the co‐occurrence of *S. sedoides* and *S. prenja* in the Apennines. They all occur at subalpine to alpine elevations and grow on limestone and dolomitic scree. As shown in detail by Hörandl (1993), the niche of *S. hohenwartii* is much narrower than that particularly of *S. sedoides*. *Saxifraga hohenwartii* as sister to *S. sedoides* + *S. prenja* is likely to have split allopatrically from the latter two species. The species has been interpreted as a relict endemic by Hörandl (1993), and its current range lies in the ‘southeasternmost calcareous Alps’ refugium for calcicole taxa (Tribsch & Schönswetter, 2003). Considering the extant ranges of *S. sedoides* and *S. prenja*, their last common ancestor most likely attained a wide range including areas outside the Alps, and the two species most likely originated by vicariance. Equally, *S. sedoides*, by occurring in the southern and northeastern Alps, a disjunction long known from other taxa (Merxmüller, 1952), and also in the Apennines, must have had a wider range in the past. The same applies to *S. prenja* which also occurs in the Apennines. Long‐distance dispersal to the Apennines of both *S. sedoides* and *S. prenja*, however, cannot be excluded. The subrange of *S. sedoides* in the northeastern Alps lies in the ‘northeastern calcareous Alps’ refugium for calcicole taxa (Tribsch & Schönswetter, 2003). Clade 2 (except for *S. prenja*) essentially is distributed allopatrically with clade 1 in the south and the remainder of the subsection in the north (Figure 1). The three species of clade 3, *S. muscoides*, *S. presolanensis* and *S. tenella*, are also distributed allopatrically. They differ strongly in ecology. Whereas the calcifuge *S. muscoides*, not resolved as monophyletic in our phylogenetic tree, is a distinctly alpine species by occurring at elevations between 2250 and 4200 m (Kaplan, 1995; Webb & Gornall, 1989), *S. tenella* grows at between 700 and 2000 m on shady limestone rocks and screes, and *S. presolanensis* grows on north‐facing and shady vertical or even overhanging limestone cliffs (Kaplan, 1995; Merxmüller & Wiedmann, 1957; Webb & Gornall, 1989) between 1800 and 2100 m. The range of *S. tenella* lies, as that of *S. hohenwartii*, in the ‘southeasternmost calcareous Alps’ refugium for calcicole taxa (Skubic et al., 2018; Tribsch & Schönswetter, 2003), and that of *S. presolanensis*, located on the very edge of the main range of *S. muscoides*, in the ‘Alpi Bergamasche’ refugium for calcicole taxa (Tribsch & Schönswetter, 2003). Although the range of *S. tenella* is widely disjunct with the major part of the range of *S. muscoides* in western parts of the Alps, the disjunct subrange of *S. muscoides* in the Tauern somewhat connects the two species. With the exception of *S. tenella*, clade 3 clearly is allopatric with clades 1, 2 and 4. Although there is a broad overlap, at a large scale, of *S. muscoides* with *S. aphylla* of clade 4, these two species are well separated by their different bedrock requirements. Finally, in clade 4, excluding *S. facchinii* as a species of hybrid origin, *S. aphylla* and *S. arachnoidea* are distributed allopatrically and are strongly differentiated ecologically. Whereas *S. aphylla* grows on calcareous scree and stony ground, often where the snow lies late, at elevations between 1730 and 3200 m (Kaplan, 1995) but mostly between 2100 and 2800 m (Webb & Gornall, 1989), *S. arachnoidea* grows between 600 and 1850 m in limestone dust under overhanging rocks (Pitschmann & Reisigl, 1959) sheltered from rain and sun (Webb & Gornall, 1989). Accordingly, the light requirements (Landolt et al., 2010) of *S. arachnoidea* (half‐shady) are much lower than those of *S. aphylla* (very bright). *Saxifraga arachnoidea* is allopatric with the more widely distributed *S. aphylla*, and its range lies within the ‘Monte Baldo, Monti Lessini, and Prealpi Bellunesi’ refugium for calcicole taxa (Schönswetter et al., 2005; Tribsch & Schönswetter, 2003).

In summary, the distribution of the four clades recognized and the distribution of species within these four clades is largely para‐ and allopatric.

### 4.2 *Saxifraga facchinii*, a hybrid species

As evident from our analyses, *S. facchinii* originated through hybridization between the last common ancestor of *S. presolanensis* and *S. muscoides* on the one hand and the last common ancestor of *S. aphylla* and *S. arachnoidea* on the other hand (Figure 2). Although a hybrid origin of *S. facchinii* had never been suspected, its relationship to *S. muscoides* is reflected in its treatment as a variety of that species by Engler (1872), and a close relationship between *S. facchinii* and *S. aphylla* had been found by Vargas (2000). Gerschwitz‐Eidt and Kadereit (2020) found the species to be closest relative of *S. muscoides*/*S. presolanensis* in their ITS phylogenetic tree, and to be closest relative (unsupported) of one sample of *S. aphylla* in their plastid phylogenetic tree. *Saxifraga facchinii* grows on calcareous rocks and scree at alpine elevations between 2250 and 3000 m and in this respect is ecologically comparable to *S. aphylla* and *S. muscoides* of its two parental lineages. Accordingly, this hybridization event did not result in major changes in ecological preferences of the resulting hybrid species.

The distribution of *S. facchinii* on the one hand and its two parental lineages on the other hand is allopatric, as very commonly observed in both homoploid and allopolyploid hybrid species (Kadereit, 2015). Following Kadereit (2015), this may be the result of the segregation of ranges of ecologically differentiated entities in response to climatic changes. Considering the distribution of *S. facchinii*, it seems possible that its parental lineages were sympatric in the area of the extant range of *S. facchinii* at a time when the range of the last common ancestor of *S. muscoides* included the major range of this species in the west and the disjunct subrange of this species in the Tauern (Figure 1). In this case the hybrid species might have persisted in its area of origin and the parental lineages migrated tracking their niches in times of climatic change. As the chromosome number of *S. facchinii* is unknown, we do not know whether the species is a homoploid or polyploid hybrid species.

### 4.3 The ancestral ecology of subsection *Arachnoideae*

Subsection *Arachnoideae* is part of section *Saxifraga* (Ebersbach et al., 2017; Tkach et al., 2015) together with subsection *Saxifraga*, subsection *Androsaceae* and *S. irrigua*. Subsection *Saxifraga* is found mainly in the Iberian peninsula, subsection *Androsaceae* mainly in the Alps, and *S. irrigua* is from Crimea, where the species grows in rocky woods and on shady cliffs, always on limestone, between 500 and 1250 m elevation (Webb & Gornall, 1989). Relationships among these three well‐supported clades and *S. irrigua* were not resolved in the phylogenetic tree of Tkach et al. (2015), although Tkach et al. (2019) obtained support for a sister relationship between *S. irrigua* and subsection *Androsaceae*. As reconstruction of the ancestral ecology of subsection *Arachnoideae* requires an outgroup, our reconstruction used part of our species tree based on Hybseq sequences (Figure 2) which altogether included nine species of subsection *Saxifraga* and resulted in a non‐monophyletic subsection *Saxifraga*. In our reconstruction, the stem group node of subsection *Arachnoideae* was reconstructed as subalpine, neutral to slightly alkaline soils and (just below) bright (Figure 5). Accordingly, the calcifuge ecology of *S. paradoxa* (and *S. muscoides*) and the reduced light requirement of *S. berica*, *S. paradoxa* and *S. arachnoidea* evolved twice independently each within the subsection. With respect to elevational distribution, our results imply that high elevation (subalpine) rather than low elevation distribution is ancestral in the subsection. However, alpine distributions evolved twice independently (*S. aphylla*, *S. muscoides*), as known from other examples (Trucchi et al., 2017).

### 4.4 Origin of divergent ecologies through adaptive evolution in refugia ‐ a hypothesis

The definition of a refugium as an area where a particular species survived for an entire glacial–interglacial cycle (Hewitt, 2004) appears to suggest that such areas were sanctuaries where species were preserved from extinction (Nieto Feliner, 2011). However, it is widely appreciated that evolutionary change resulting in speciation took place in the Quaternary through geographical isolation in refugia and through hybrid speciation upon secondary contact (Kadereit & Abbott, 2022). Evolutionary change most likely also took place in response to exposure to novel selection pressures in biotic and abiotic conditions, different from the conditions in areas where the species lived and went extinct in glacial periods of the Quaternary (Davis & Shaw, 2001; De Lafontaine et al., 2018; Nieto Feliner, 2011; Stewart et al., 2010; Stewart & Stringer, 2012). Abiotic and biotic conditions in refugia changed through Quaternary times. Considering alpine species, the glacial refugia in the area of the European Alps identified by Schönswetter et al. (2005) were cold‐stage refugia (Birks & Willis, 2008). In interglacials or the Holocene, both changing climatic conditions and competition in the former cold‐stage refugia in most cases will have resulted in the re‐migration of alpine species into high elevation habitats, i.e., into their warm‐stage refugia (Birks & Willis, 2008). Considering the extant distribution of *S. berica*, *S. paradoxa* and *S. arachnoidea* in well‐known refugial areas, and assuming that these refugia, identified for the Last Glacial Maximum (Schönswetter et al., 2005; Tribsch & Schönswetter, 2003), can be taken as proxies for the location of cold‐stage refugia in earlier parts of the Quaternary, we here hypothesize that the extant ecology of these species originated through adaptive evolution in refugial areas where they persisted irrespective of climatic change. *Saxifraga paradoxa* is one of two calcifuge species of the subsection by growing on gneiss or mica‐schists. We consider it highly likely that the shift from calcicole to calcifuge followed the climatically enforced migration of a calcicole ancestor into a cold‐stage refugium for calcifuge plants. Shifts from calcicole to calcifuge and vice versa linked to and likely resulting from Quaternary migration and dispersal into edaphically different areas had before been postulated for *Adenostyles* Cass. (Dillenberger & Kadereit, 2013) and *Jovibarba* (DC.) Opiz and *Sempervivum* L. (Klein & Kadereit, 2015).

The low light requirements of *S. berica*, *S. paradoxa* and *S. arachnoidea* are likely to have evolved in response to changing environmental conditions in their respective distribution ranges. These three species all have very small distribution ranges which lie within well known cold‐stage refugia (Schönswetter et al., 2005). Accordingly, they are likely to have persisted in their refugia. Considering that cold‐stage refugia on the edges of the Alps most likely contained more or less all habitat types of extant alpine species, we consider it likely that the extant habitats of *S. berica*, *S. paradoxa* and *S. arachnoidea* do not correspond to those encountered when the populations which gave rise to these species initially migrated into cold‐stage refugial areas. Instead, occupation of these very marginal habitats with little interspecific competition probably is the result of competitive exclusion from other sites after the recolonization of cold‐stage refugial areas by more competitive species in interglacial times. All three species, as indeed all species of subsection *Arachnoideae*, were assessed as stress‐tolerators (vs. competitors; Grime, 1974) by Landolt et al. (2010). Interestingly, the habitats of *S. berica*, *S. paradoxa* and *S. arachnoidea* somewhat resemble some of those habitats occupied by extant alpine species when growing at low elevations. These, in the British Isles, include screes, north‐facing slopes, steep cliffs and cool ravines (Birks & Willis, 2008; Pigott & Walters, 1954), and gorges in the Mediterranean area (Gentili et al., 2015). Whereas the ecology of extant alpines at low elevations or southern latitudes is, as far as known, the result of competition and thus of ecological processes, we hypothesize that the limitation of *S. berica*, *S. paradoxa* and *S. arachnoidea* to their specific habitats is the result of adaptation and thus of evolutionary processes. Adaptation to conditions into which species were forced by competition before has been postulated for an eastern North American narrow endemic of *Dodecatheon* L. limited to patchy cool cliffs by Oberle and Schaal (2011) and the serpentine endemic *Cherleria* (= *Minuartia* L.) *laricifolia* (L.) Iamonico subsp. *ophiolitica* (Pignatti) Iamonico from the northern Apennines by Moore et al. (2013). Interestingly, Webb and Gornall (1989) observed that cultivation of *S. berica*, *S. paradoxa* and *S. arachnoidea* is only possible when the conditions of their natural habitat, particularly shade, are reproduced as closely as possible. This clearly implies that these three species are not limited to their present niches by competition alone but require the conditions found for successful growth and reproduction. It remains unclear whether these marginal habitats were colonized only as the result of increasing competition or had been part of the niche of these species before. In the first case, novel selection pressures resulting in evolutionary divergence thus not only arose from abiotic environmental conditions but probably mainly from interspecific competition, the ‘new neighbors’ of Hewitt (1996, 2000, 2004). When considered together with their closest relatives, *S. berica* (closest relatives: *S. petraea*, *S. paradoxa*), *S. paradoxa* (*S. petraea*) and *S. arachnoidea* (*S. aphylla*), persisting in cold‐stage refugial areas upon climatic warming, can be considered stable rear edge populations or species (Hampe & Petit, 2005). For such populations, Ackerly (2003) proposed, in his *‘trailing edge hypothesis of adaptive evolution*’, that the most important condition for adaptive evolution to occur at the rear edge is the exclusion of competitors. In case of *S. berica*, *S. paradoxa* and *S. arachnoidea* this appears to have been realized by the evasion of competitors through adaptation to shady habitats unsuitable for them.

If indeed adaptation to novel selection pressures in *S. berica*, *S. paradoxa* and *S. arachnoidea* took place in warm stages of the late Pliocene and Pleistocene, as also postulated for adaptive differentiation within *Taxus baccata* L. (Mayol et al., 2015), geological time available for evolutionary change was even shorter than in cold stages because warm stages were substantially shorter than cold stages in the Pleistocene (Birks, 2019). Although it has been argued that periods of isolation required for speciation were never long enough in the Quaternary (Willis & Niklas, 2004), speciation in Quaternary glacials has been implied by Kadereit et al. (2004). For *S. berica*, *S. paradoxa* and *S. arachnoidea* we do not postulate that they originated in interglacial times. Instead, they are more likely to have originated when pushed into refugia in late Pliocene/Pleistocene cold stages. However, their extant ecological make‐up appears to be the result of adaptive evolution in Quaternary interglacials. In consequence, both Late Pliocene/Pleistocene cold stages, resulting in geographical isolation, and warm stages, inducing adaptive change, shaped the evolution of these species.

## AUTHOR CONTRIBUTIONS

**Joachim Walter Kadereit:** Conceptualization (lead); formal analysis (supporting); funding acquisition (lead); writing – original draft (lead); writing – review and editing (lead). **Markus S. Dillenberger:** Formal analysis (supporting); methodology (supporting); writing – original draft (supporting); writing – review and editing (supporting). **Michael A. Gerschwitz‐Eidt:** Data curation (lead); formal analysis (lead); methodology (lead); project administration (lead); visualization (lead); writing – original draft (supporting); writing – review and editing (supporting).

## CONFLICT OF INTEREST

The authors declare we have no conflict of interest.

## Data Availability

The data supporting the findings of this study are available at the NCBI Sequence Read Archive at https://www.ncbi.nlm.nih.gov/sra, BioProject accession number PRJNA702656.

## APPENDIX A

**Method 1: exon paralog filtering**

During sequence assembly, multiple contigs per locus were assembled for almost all loci and samples of *Saxifraga* sect. *Saxifraga*. Those contigs might either be true alleles or paralogs. A three‐step procedure was used to separate orthologous (i.e., alleles) and paralogous sequences. First, the quality‐filtered DNA reads were assembled with HybPiper using the original sequence reference from Folk et al. (2019). Subsequently, the HybPiper post‐processing scripts were used as described in Johnson *et al*. (2016) to identify orthologs and paralogs in *Saxifraga* sect. *Saxifraga*: The HybPiper scripts 'paralog_investigator.py' and 'paralog_retriever.py' were used to extract the exonic sequences of all contigs at each locus, to align them for all samples using MAFFT v7.305 (Katoh & Standley, 2013) and to calculate a phylogenetic maximum likelihood (ML) gene tree from each alignment. The gene trees were then visually evaluated for the presence of paralogous sequences. Putative paralogous sequences were identified in the ML trees based on the segregation pattern of multiple sequences of the same sample: Assuming a specimen is represented by multiple orthologous sequences in the sequence dataset of a phylogeny, the leaves of this individual in the phylogenetic tree either form a monophylum (in the absence of incomplete lineage sorting; ILS) or are at least close relatives (under low ILS). Paralogous sequences, on the other hand, are not closely related in the gene tree but instead group with the respective orthologous sequences of other samples. For all loci where paralogs were identified by this rule, two to four sequences from each group of putative orthologs were selected and added to an updated reference sequence file as new reference sequences for their respective ortholog. Previous sequence references for these loci were deleted so that, in a second DNA assembly, sequences from these loci could be assembled exclusively against the updated reference sequences. Using this updated reference sequence file, DNA assembly with HybPiper and post‐processing steps was repeated as described above. Paralog assessment was repeated as described above and additional reference sequences from putative orthologs were added to an updated reference file. Finally, after a third DNA assembly, all loci still containing paralogs in samples of *Saxifraga* subsect. *Arachnoideae* were discarded. For the remaining loci, all further steps of HybPiper were performed to also obtain the non‐exonic sequence data.

**Method 2: non‐exon paralog filtering**

We used TreeShrink v1.3.3 (Mai & Mirarab, 2018) to identify individual samples or clades that contributed disproportionately to the length of a phylogenetic tree by containing, e.g., few paralogs in an otherwise mostly orthologuous dataset, and to automatically remove the corresponding DNA sequences from the alignments. First, we calculated ML gene trees with RAxML v8.2.12 (Stamatakis, 2014) using an unpartitioned GTR + Γ nucleotide substitution model with *S. hirsuta* as outgroup. Second, all gene trees and alignments were analyzed together in a single TreeShrink run to remove outlier sequences. Alignments were finally re‐examined with BuddySuite v1.3.0 to trim all alignment positions that consisted of more than 50% gaps, and to remove all alignments missing more than 20% of the samples or the *S. hirsuta* sample.

**Method 3: phylogenetic network bootstrap analysis**

Phylogenetic networks were calculated using SNaQ (Solís‐Lemus & Ané, 2016) as implemented in PhyloNetworks v0.11.0. First, we created a table of quartet concordance factors (Cfs) in PhyloNetworks, using 388 bootstrapped ML gene trees, calculated in RAxML v8.2.12, for all combinations of four different taxa each. Six phylogenetic MPL networks were then iteratively calculated from the Cfs table in SNaQ guided by a taxon map for zero to five hybrid nodes (H). The reconstruction was initialized for H = 0 using the ASTRAL species tree as the starting tree. The networks for H = 1–3 were each initialized with the best network from the previous run. Network reconstruction for H = 4 could not be initialized with the best network for H = 3 because no further reticulation edges could be added to it without the creation of intersecting cycles which are prohibited in SNaQ. Instead, the calculations of the networks for H = 4 were initialized with the best network for H = 2. For the same reasons, neither the best network for H = 3 nor for H = 4 could be used to initialize the calculation of the network for H = 5. Therefore, it was also initialized with the best network for H = 2. For each value of H=0–5, 50 independent network calculations were performed. The pseudo‐likelihoods of the best networks for each value of H were plotted against H and the shape of the curve was interpreted to determine the most likely number of hybrid nodes and thus the most likely network.

Bootstrap analyses were performed in SNaQ for the best networks of H = 1 and H = 2 to incorporate gene tree estimation error into the network calculation (Solís‐Lemus, personal communication 2020). The 38,800 bootstrap replicates of the 388 ML gene trees were used as input data. First, one random gene tree bootstrap replicate was selected for each of the 388 gene trees. Second, a table of quartet Cfs was generated from these 388 gene tree bootstrap replicates. Third, the best network was reconstructed from the Cf table in 50 independent runs to obtain a single network bootstrap replicate. Fourth, this process was performed a total of 100 times each for H = 1 and H = 2 to generate two bootstrapped networks with 100 bootstrap replicates each.

**Method 4: locus selection for divergence times estimation**

We used the full data set of 58 samples to identify several loci for which gene trees could be unambiguously assigned to one of the two topologies contained in the multi‐labelled tree as inferred from the 42 samples network reconstructions. We aimed to select a total of 20 gene trees, with ten trees for each of the two topologies. To achieve this, we first calculated Robinson‐Foulds (RF) distances of the gene trees to the two species tree topologies in PhyloNetworks v0.11.0 for each of the 405 gene trees of the 58 samples data set. For the calculation of RF distances to the first topology, we used the original 58‐samples species tree calculated with ASTRAL (Figure A1). For the calculation of RF distances to the second topology, the ASTRAL species tree was permuted by moving the *S. facchinii* clade from clade 4 to clade 3. We identified all gene trees that had different RF distances to the two species tree topologies. These gene trees were visually inspected to determine whether their topologies could each be unambiguously assigned to one of the two topologies contained in the multiple‐labelled phylogenetic species tree. Nodes with bootstrap support less than 70% were interpreted as polytomies in this process. Using these criteria, 10 loci with a taxon sample as complete as possible were selected for the divergence times estimation for each of the two species tree topologies.
