# Corrigendum

**DOI:** 10.1002/ece3.9780

**Published:** 2023-02-15

In the recent article by Gerschwitz‐Eidt et al. (2023), the authors would like to correct the sequence of the Figures A1, A2, A3 as follows:

**FIGURE A1:**
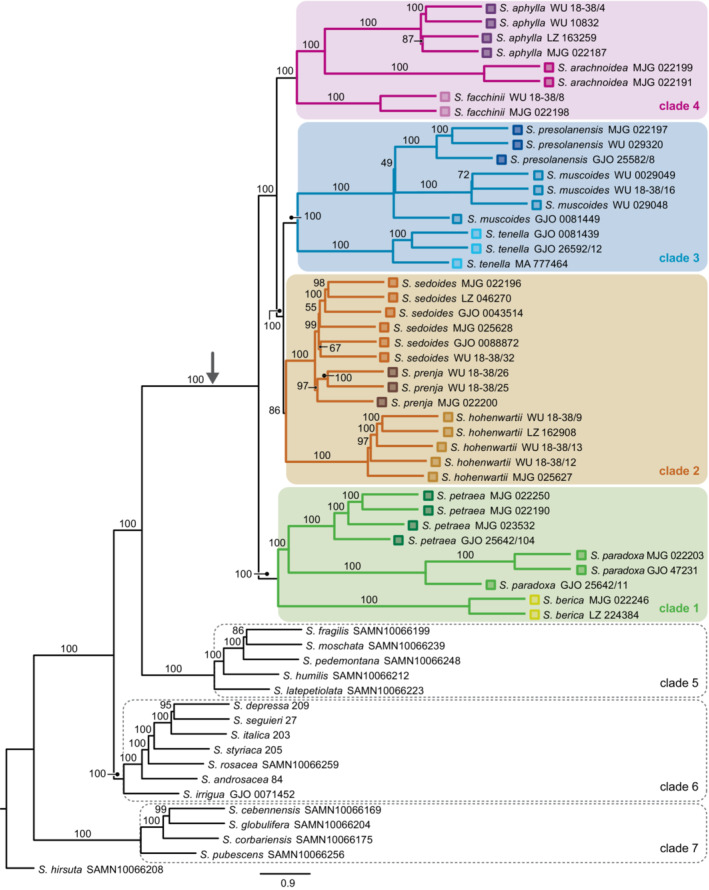
Summary coalescence species tree calculated in ASTRAL. The stem branch of subsection *Arachnoideae* is marked with an arrow.

**FIGURE A2:**
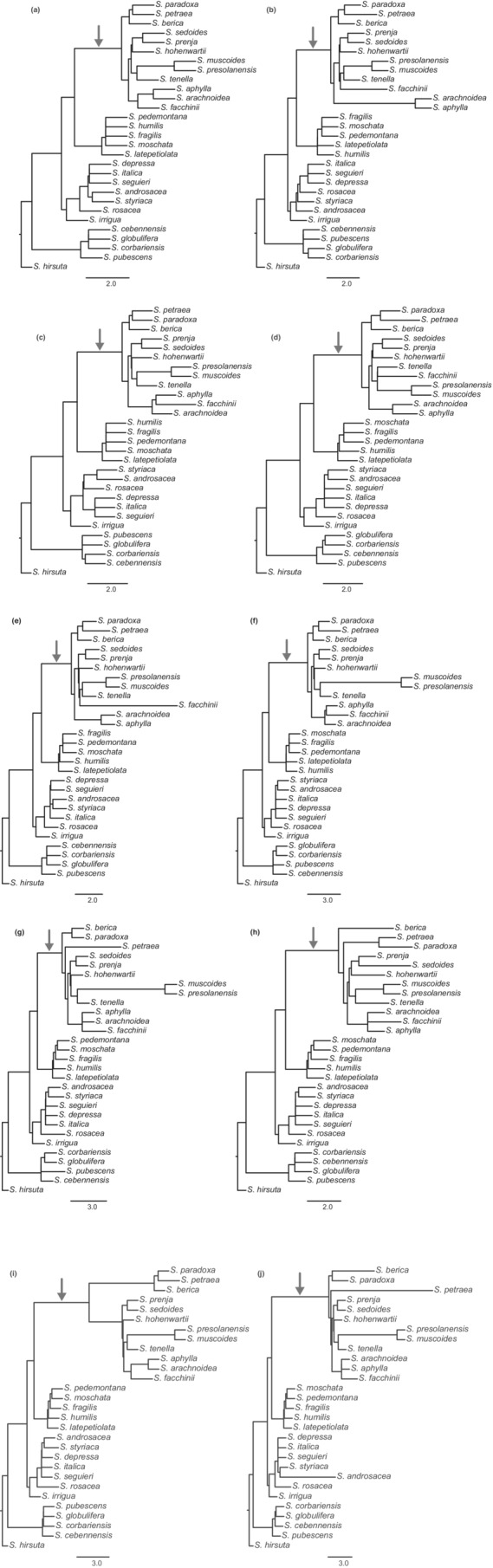
Cladograms of the major trees of 11 maximum pseudo‐likelihood phylogenetic networks calculated in PhyloNet. The stem branch of subsection *Arachnoideae* is marked with an arrow.

**FIGURE A3:**
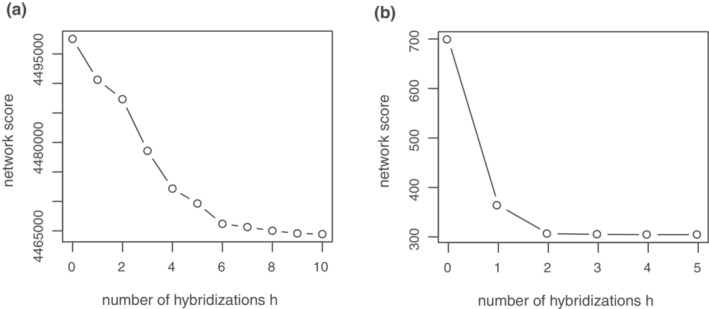
Log pseudo‐likelihood profiles for phylogenetic networks with (a) 58 and (b) 42 samples for different numbers of reticulate nodes.

The authors apologize for the error.
